# Direct leaf-peeling method for areca protoplasts: a simple and efficient system for protoplast isolation and transformation in areca palm (*Areca catechu*)

**DOI:** 10.1186/s12870-023-04048-7

**Published:** 2023-01-26

**Authors:** Yaodi Wang, Linxi Wang, Hongjun Liu, Bei Gou, Weiyao Hu, Li Qin, Wentao Shen, Aiming Wang, Hongguang Cui, Zhaoji Dai

**Affiliations:** 1 Sanya Nanfan Research Institute, Key Laboratory of Green Prevention and Control of Tropical Plant Diseases and Pests (Ministry of Education), School of Plant Protection, Hainan University, Haikou, Hainan 570228 China; 2 Key Laboratory of Biology and Genetic Resources of Tropical Crops, Ministry of Agriculture and Rural Affairs & Institute of Tropical Bioscience and Biotechnology, Chinese Academy of Tropical Agricultural Sciences, Haikou, Hainan 571101 China; 3 London Research and Development Centre, Agriculture and Agri-Food Canada, 1391 Sandford Street, London, ON N5V 4T3 Canada

**Keywords:** Areca palm, *Areca catechu*, Protoplast, Protoplast isolation, PEG-mediated transformation

## Abstract

**Background:**

Areca palm (*Areca catechu*) is a woody perennial plant of both economical and medicinal importance grown in tropical and subtropical climates. Yet, the molecular biology study of areca palm is extremely impeded by its unavailability of a transformation method. An efficient protoplast isolation and transformation system could be highly desirable to overcome this barrier.

**Results:**

Here, we described a simple and efficient method for protoplast isolation and transformation from the perennial plant areca palm. A high yield of protoplasts (2.5 × 10^7^ protoplasts per gram of fresh leaf tissues) was obtained from the fresh light green leaflet from the newly-emerged leaf digested overnight in the enzyme solution [2% (w/v) cellulase R10, 0.5% (w/v) macerozyme R10, 0.7 M mannitol, 10 mM CaCl_2_, 20 mM KCl, 20 mM MES and 0.1% (w/v) BSA, pH 5.7] by the direct leaf-peeling method. The isolated areca protoplasts maintain viability of 86.6% and have been successfully transformed with a green fluorescent protein (GFP)-tagged plasmid (pGreen0029-GFP, 6.0 kb) via the polyethylene glycol (PEG)-mediated transformation. Moreover, the mannitol concentration (optimal: 0.7 M) was determined as a key factor affecting areca protoplast isolation. We also demonstrated that the optimal density of areca protoplast for efficient transformation was at 1.0–1.5 × 10^6^ cells/ml. With the optimization of transformation parameters, we have achieved a relatively high transformation efficiency of nearly 50%.

**Conclusion:**

We have established the first efficient protocol for the high-yield isolation and transformation of areca palm protoplasts. This method shall be applied in various biological studies of areca palm, such as gene function analysis, genome editing, protein trafficking and localization and protein–protein interaction. In addition, the protoplast system offers a great genetic transformation approach for the woody perennial plant-areca palm. Moreover, the established platform may be applied in protoplast isolation and transformation for other important species in the palm family, including oil palm and coconut.

## Background

Areca palm (*Areca catechu*), a species in the palm family (Arecaceae), is a perennial plant of both economical and medicinal importance grown in much of the tropical Asia, Pacific and parts of Africa [1, 2]. In China, areca palm is grown in tropical and subtropical regions, including Hainan, Taiwan, Yunnan and Guangxi provinces [3]. In the past few years, areca palm plantations continued to grow as an economic investment in Hainan, as a result, there are notable increases in areca palm biology studies, such as genomics, breeding, gene function, evolution and plant pathology [2, 4–7]. Unfortunately, compared to the other two typical species in the palm family, coconut and oil palm, the biological study of areca palm falls far behind. Yet, there is no report on transgenic areca palm, nor transformation methods for areca palm. It was not until after 2021 that two individual groups in Hainan reported a chromosome-scale genome assembly of the *A. catechu,* respectively [2, 4]. This will enable detailed studies in areca palm biology.

Plant transformation is a core tool for plant biology. The most common transformation strategies include *Agrobacterium tumefaciens*-mediated transformation, biolistic bombardment and protoplast transformation [8]. Although significant advancement in plant transformation has been developed for hundreds of plant species, areca palm is still recalcitrant to this process, making an efficient transformation highly demanded in the biology studies of areca palm [9]. Nevertheless, we have to acknowledge that protoplast isolation and transformation platforms have been established for oil palm, the same family as areca palm, despite its demerits of being time-consuming and labor-intensive (using friable embryogenic callus for protoplast isolation), and low transfection efficiency (< 5% and 20%, respectively for two reports) [10, 11]. A simple and efficient system for protoplast isolation and transformation is demanded in the palm family.

Plant protoplasts are the naked plant cells that lack the rigid plant cell wall, and serve as a well-known tool for efficient transformation [9, 12]. Successful protoplast isolation relies on the optimization of various isolation factors including starting plant material, cellulase/macerozyme concentration, mannitol concentration, enzymatic digestion time, pH value of enzyme solution, etc. The protoplast transfection system involves successful protoplast isolation from plant tissue and subsequent transfer of plasmid DNA into the protoplasts by using various methods, including polyethylene glycol (PEG)-mediated transformation, microinjection and electroporation [12–16]. This system has been broadly applied in transient gene expression such as subcellular localization, protein–protein interaction and genome editing studies [9, 12–17]. Moreover, protoplast transformation and downstream attempts can be a valuable approach for generating stable transgenic plants, especially for these plant species recalcitrant to agrobacterium-mediated transformation [8, 9].

In this work, we describe a simple and efficient system for areca palm protoplasts (henceforth areca protoplasts) isolation and PEG-mediated transformation using the fresh light green leaflet from the newly-emerged leaf as starting material. We demonstrated that the mannitol concentration and protoplast density play critical roles in protoplast isolation and transformation, respectively. In addition, this is the first study to describe protoplast isolation and transformation in areca palm. Transformed protoplasts are of significant value in areca biology, e.g. breeding, gene function studies, pathological studies and transgenic studies.

## Results

### Identifying suitable fresh leaflet material as a protoplast source from areca palm

**Fig. 1:**
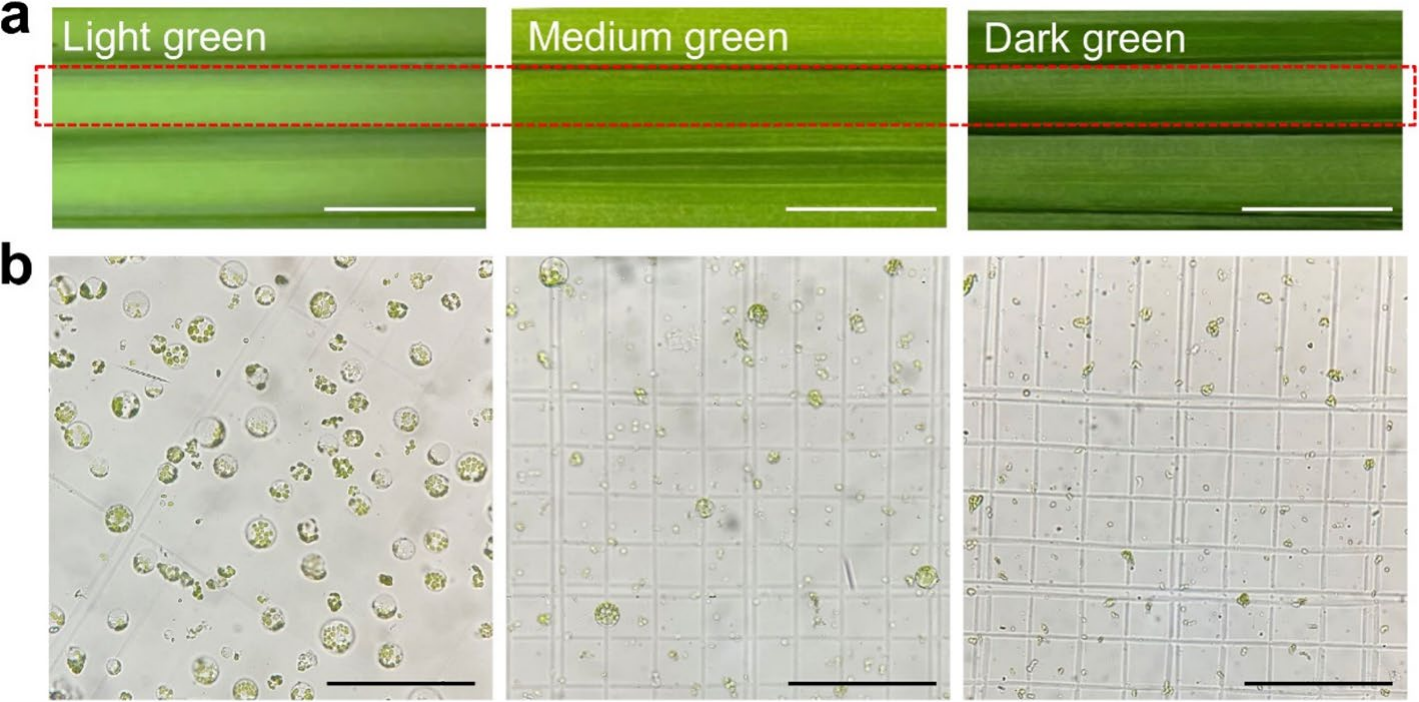
Starting leaflet materials for areca palm protoplast isolation. **a** Different fresh leaflet materials (light, medium and dark green leaflets, respectively) indicated by the red dashed rectangle from an 18-month-old areca palm plant at the three-leaf stage for protoplast isolation. The red dashed rectangle indicates the areas between leaf veins for distinguishing three different greens. Scale bar = 1 cm; **b** Released protoplasts after incubation with enzyme solution overnight. Scale bar = 100 μm

Considering current protoplast isolation and transformation of oil palm requires calli formation through tissue culture which is time-consuming and labor-intensive [10, 11], our first aim is to search for a simple and efficient method for areca protoplast isolation. To test the accessibility of areca leaflets for protoplast isolation, an 18-month-old areca plant at the three-leaf stage was selected. We investigated different starting leaflet materials as the protoplast sources: light green leaflet from the 3rd leaf (the newly emerged leaf), approximately 7 days after 3rd leaf emergence; medium green leaflet from the 2nd leaf and dark green leaflet from the 1st leaf (red dashed rectangle) (Fig. 1a). Upon the digestion process using the enzyme solution, a large number of intact protoplasts (176 protoplasts per field) were observed using a hemocytometer under the microscope in the light green leaflet sample. In contrast, very few (only 14 protoplasts per field) were detected in the medium leaflet sample and no protoplasts were observed in the dark leaflet sample (Fig. 1b). These data suggest that the light green leaflet from the newly-emerged leaf serves as an excellent source for areca protoplast isolation.

### Mannitol concentration plays a major role in areca protoplast isolation

**Fig. 2:**
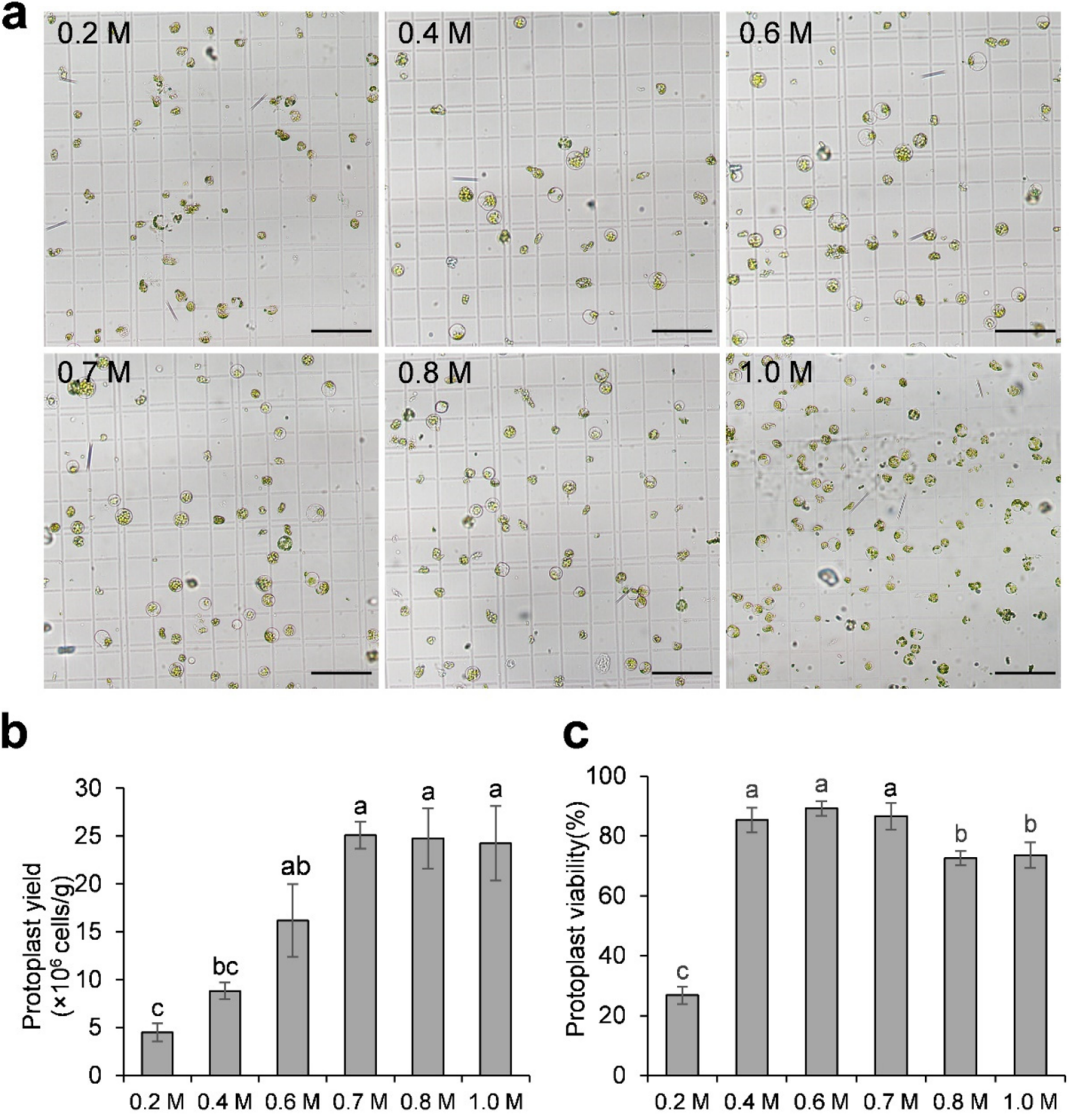
Effects of mannitol concentration on areca palm protoplast isolation. **a** Released protoplasts on a hemocytometer. Mannitol concentration was set at 0.2, 0.4, 0.6, 0.7, 0.8 and 1.0 M, respectively. Released protoplasts after enzyme solution digestion overnight were visualized under a light microscope. Scale bar = 100 μm; **b** Effect of mannitol concentration on areca protoplast yield. The number of round and intact protoplasts was counted under a light microscope. Error bars represent the *SD* of three replicates. Different lowercase letters represent significant statistical difference. **c **Viability test for protoplasts isolated with different mannitol concentrations. At least 80 protoplasts exhibiting green fluorescence under a fluorescent microscope were counted. Error bars represent the *SD* of four replicates. Different lowercase letters represent significant statistical difference

As the concentration of mannitol in the enzyme solution is critical for protoplast integrity [17], we tested a series of different concentrations of mannitol in the enzyme solution for protoplast release from the areca palm leaflet. We found that mannitol at 0.7 M resulted in the highest yield (2.5 × 10^7^ protoplasts per gram) with decent integrity, followed by 0.8 M as the second highest (Fig. 2a, b). In contrast, mannitol concentration at lower concentrations of 0.2 M and 0.4 M resulted in poor protoplast yield (Fig. 2a, b). Furthermore, a higher concentration at 1.0 M led to a negative impact on the integrity of protoplasts, despite the comparable protoplast yield (Fig. 2a, b). At 0.7 M mannitol, the protoplast viability is 86.6%, however, protoplast viability decreased to 72.6% and 73.6% as mannitol concentration rose from 0.7 M to 0.8 M and 1.0 M, respectively (Fig. 2c). Hence, 0.7 M mannitol was the most suitable for areca protoplast isolation in the present experiment. Taken together, these data revealed that mannitol concentration in the enzyme solution plays an essential role in efficient areca protoplast isolation.

### Direct leaf-peeling method for protoplast isolation from areca palm leaf

**Fig. 3:**
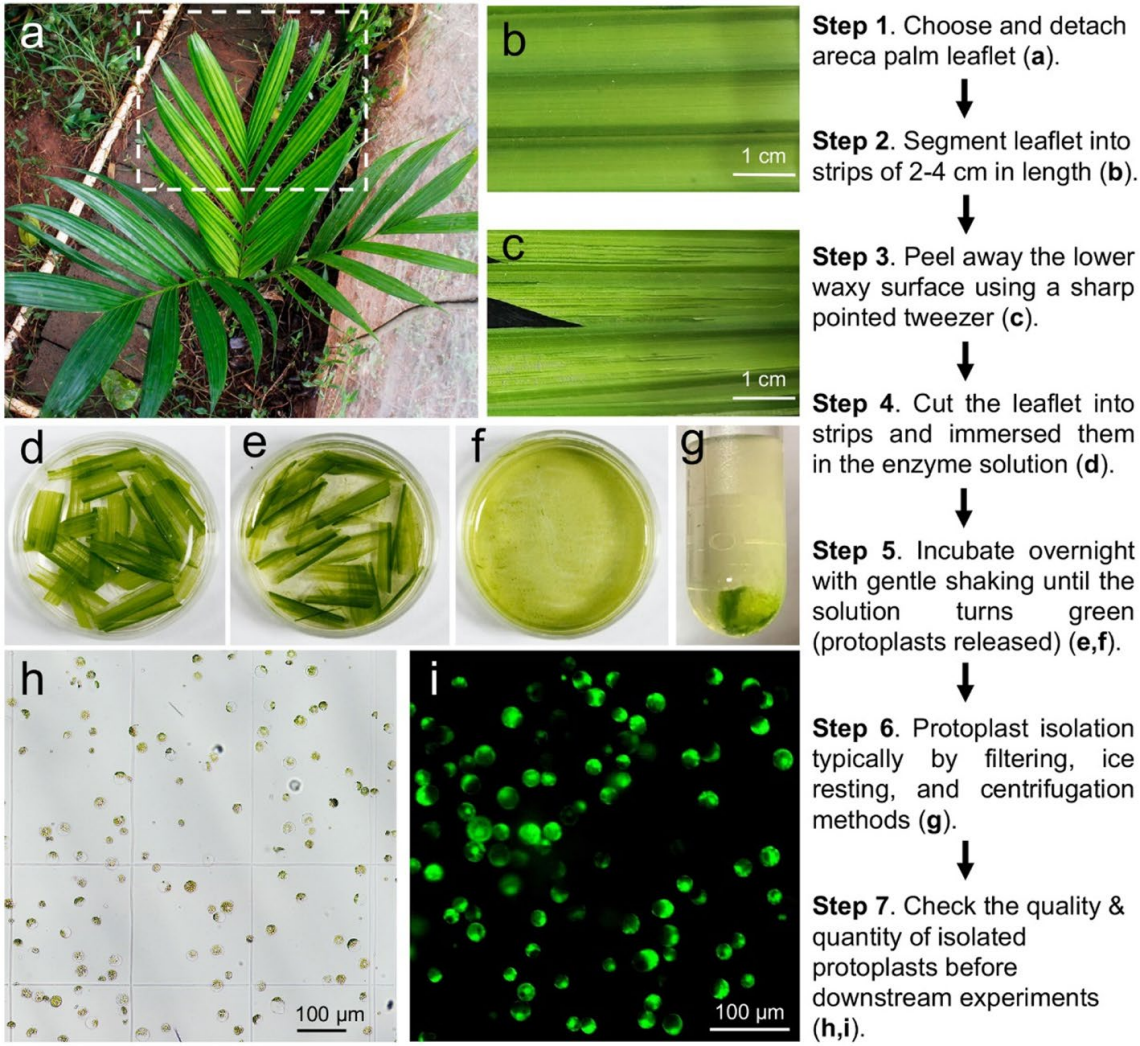
Schematic illustration of areca palm protoplast isolation. **a** A healthy 18-month-old areca palm plant suitable for protoplast isolation. Dashed-line box indicates the optimal leaflets (light green leaflet of the newly emerged leaf) to be used as the mesophyll protoplast source; **b** Selected leaflet was cut into 2–4 cm long for peeling; **c** Areca palm leaflet after peeling the waxy surface using a sharp pointed tweezer; **d** Peeled leaflet sections incubated in the enzyme solution for releasing protoplasts; **e** Protoplasts released in enzyme solution after overnight incubation; **f** Green solution containing released protoplasts in a petri dish; **g** Pelleting protoplast through optimized low-speed centrifugation; **h** Isolated areca palm protoplasts resting in MMg solution; **i**. Protoplast viability test using FDA staining

After the successful selection of leaflets and optimization of mannitol concentration for protoplast isolation, we have developed a simple and efficient method called the “Direct Leaf-Peeling Method” for areca palm protoplast isolation. In this, the light green leaflet from the newly-emerged leaf of a 12–30-month-old plant was selected (Fig. 3a) and segmented into parts of 2–4 cm long (Fig. 3b). The segmented leaves were then pulled the lower waxy layer away using a sharp pointed tweezer (Fig. 3c) and subsequently exposed to the enzyme solution containing cell wall-degrading enzymes [2% (w/v) cellulase R10, 0.5% (w/v) macerozyme R10, 0.7 M mannitol, 10 mM CaCl_2_, 20 mM KCl, 20 mM MES and 0.1% (w/v) BSA, pH 5.7] (Fig. 3d). After the overnight incubation, protoplasts are released and harvested by methods for the classic Arabidopsis protoplast isolation system with a few optimizations (Fig. 3e-g) [14]. The isolated areca protoplasts remain intact and viable illustrated by the fluorescein diacetate (FDA) staining (Fig. 3h, i). The protoplast viability test revealed that the average viability of protoplast in the current protocol is 86.6% (Fig. 2c) and a representative microscope image of protoplast with viability of 85.7% is presented in Fig. 3i.

### PEG-mediated protoplast transformation for areca protoplast

**Fig. 4:**
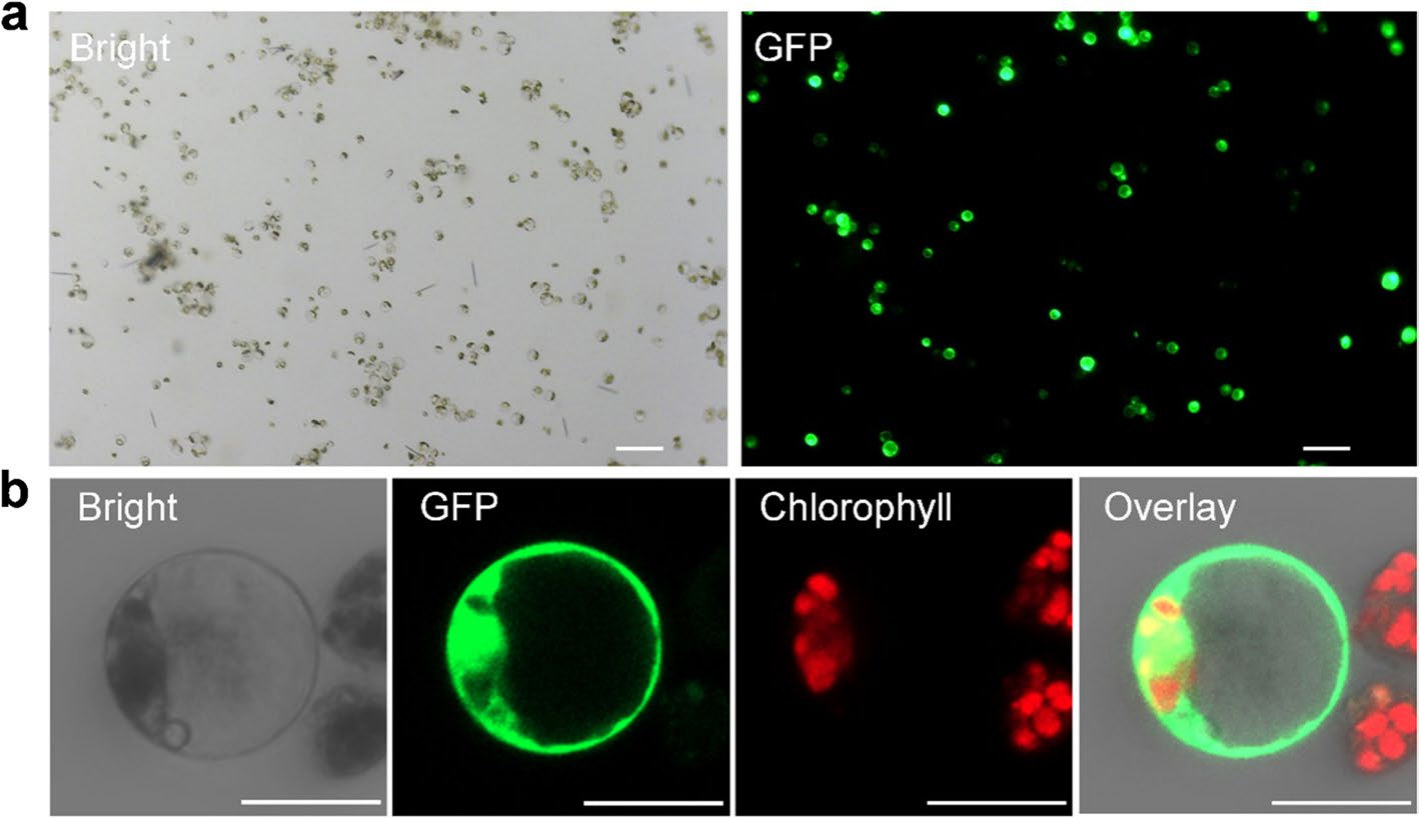
PEG-mediated transformation of areca palm protoplast. **a** PEG-mediated transformation of areca palm protoplast at 48 h post-transfection (hpt). 3 × 10^4^ areca protoplasts were transformed with 3 μg of a green fluorescent protein (GFP)-expressing plasmid (pGreen0029-GFP, 6.0 kb) and observed under a fluorescent microscope. Scale bar = 100 μm; **b** Close view of pGreen0029-GFP expression in areca protoplast at 48 hpt. Scale bar = 20 μm

For protoplast transformation, we applied the classic PEG-mediated transformation method for Arabidopsis/Tobacco [14, 15]. A green fluorescent protein (GFP)-tagged plasmid driven by the constitutive CaMV35S promoter (pGreen0029-GFP, 6.0 kb) was successfully delivered into areca protoplast using PEG-mediated transformation (Fig. 4a). GFP expression was visualized at 48 h-post transfection (hpt) using a confocal microscope and detected both in the nucleus and cytoplasm (Fig. 4b), consistent with its subcellular localization in Arabidopsis/tobacco leaf protoplast and intact plant cells. We have to point out that GFP expression can be detected as early as 12 hpt and we consistently observed higher transfection rates at 48 and 72 hpt, respectively, compared to that of 12 hpt.

### Effect of protoplast density on PEG-mediated transformation of areca protoplasts

**Fig. 5:**
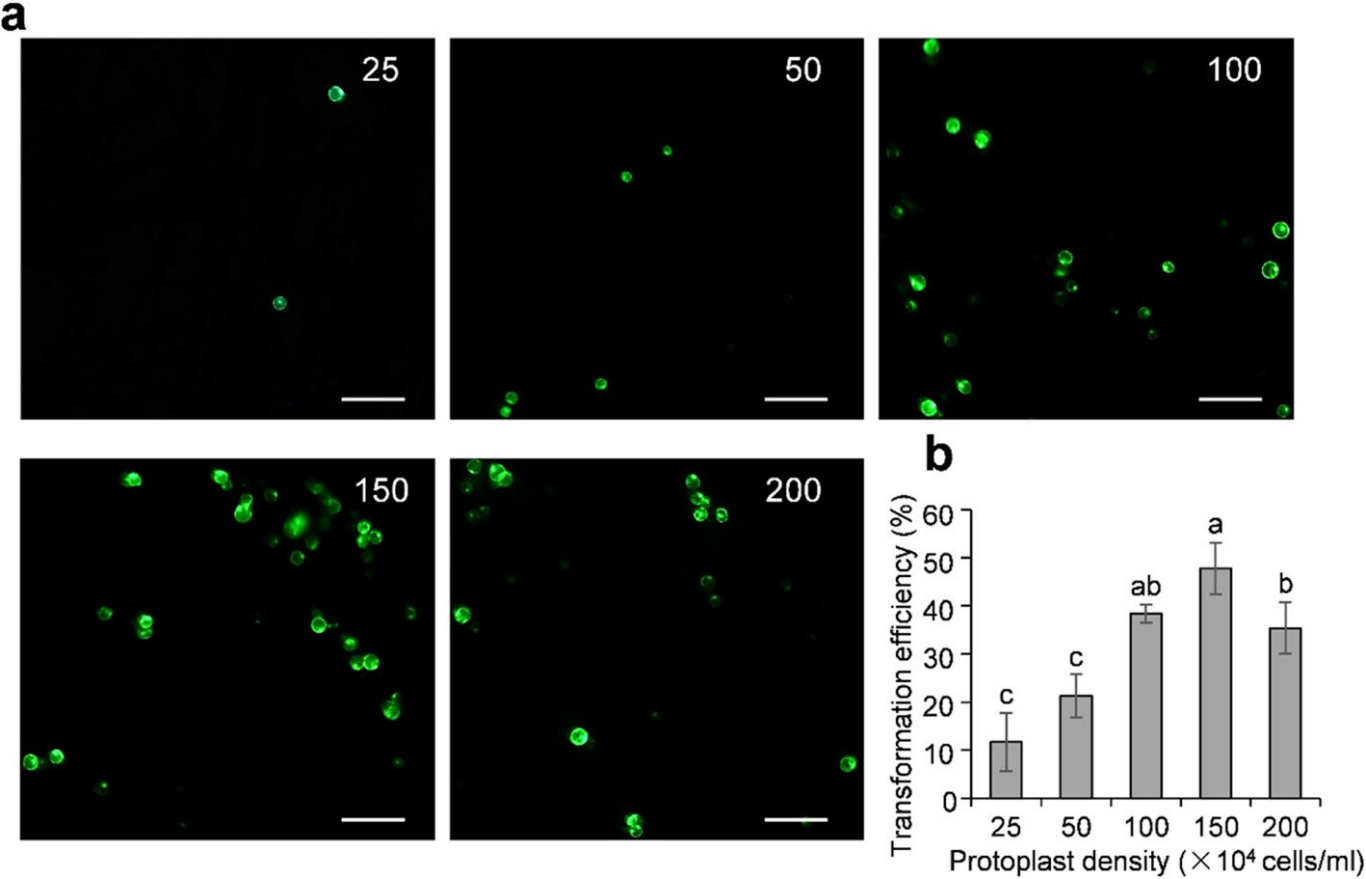
Effect of protoplast density on PEG-mediated transformation of areca palm protoplasts. **a** GFP imaging of areca palm protoplasts transformed with pGreen0029-GFP at various cell densities of 25, 50, 100, 150 and 200 × 10^4^ cells/ml, respectively at 48 hpt. Scale bar = 100 μm. **b** Effects of protoplast density on transfection efficiency. Areca protoplasts at various cell densities (30 μl each) were transfected with 3 μg of pGreen0029-GFP plasmid DNA, respectively. Transfection efficiency was calculated as the percentage of green fluorescent protoplasts divided by the total number of protoplasts. Error bars represent the *SD* of three replicates. Different lowercase letters represent significant statistical difference

Various transformation parameters have huge impact on transformation efficiency of protoplast, including protoplast density, plasmid amounts, and PEG concentration, etc. To achieve relative high transformation efficiency, we tested the effect of protoplast density on PEG-mediated transformation of areca protoplasts. A series of protoplast densities (25, 50, 100, 150 and 200 × 10^4^ cells/ml, respectively) was set to test the protoplast transformation efficiency. Interestingly, protoplast density at 100 and 150 × 10^4^ cells/ml resulted in a relative higher transformation efficiency of about 38% and 48%, respectively (Fig. 5), although there was no significant difference between two different densities according to the Bonferroni correction. Further increasing the protoplast density to 200 × 10^4^ cells/ml decreased transformation efficiency. In contrast, low protoplast density at 25 and 50 × 10^4^cells/ml produced poor transformation efficiency of about only 12 and 21%, respectively (Fig. 5). These results demonstrated that protoplast density plays a significant role in PEG-mediated transformation of areca protoplasts.

## Discussion

Protoplast isolation and transformation are widely used in molecular plant research, including transient expression, genome editing and plant pathology studies. This study aims to isolate protoplasts from areca palm, a woody perennial plant found in tropical and subtropical regions. We successfully isolated the protoplasts from the fresh light green leaflet of the newly-emerged leaf with high yield and viability and delivered a GFP-expressing plasmid into the protoplast using PEG-mediated transformation. To our best knowledge, this is the first report for efficient areca palm protoplast isolation and transfection. This system serves as an excellent platform for the molecular biology study of areca palm.

Currently, oil pam is the most documented species for protoplast isolation and transformation in the palm family, where researchers use the friable callus as the source material for obtaining protoplasts [10, 11]. In the present paper, fresh leaflet tissue was selected as the source for areca protoplasts isolation that avoids the efforts for friable callus availability through plant tissue culture. In addition, we achieved much higher protoplast yield using fresh leaflet tissue than that of oil palm using callus as protoplast source (2.5 × 10^7^ protoplasts VS 1.14 × 10^6^ protoplasts per gram of fresh weight) with slightly higher protoplast viability (86.6% VS 82%) [18]. Furthermore, we applied tweezers to directly peel off the waxy layer of areca palm leaflets, allowing the enzyme solution gets much easier access to the mesophyll cells and the intercellular space of leaflets. This method is simple and eliminated the process of slicing the leaf into 0.5–1-mm strips followed by vacuumizing which is broadly applied in protoplast isolation of various plant species, including the model plants Arabidopsis and tobacco [14, 15]. In this paper, the light green leaflet from the newly-emerged leaf was determined to be the optimal starting material for areca protoplast isolation. We have to clarify that the newly emerged leaf starts from light green and would continuedly to grow till medium/dark green as time goes on, our experience is that the newly emerged leaf can serve as the starting materials for areca protoplast isolation as long as it appears light green.

In general, the optimal mannitol concentration for protoplast isolation ranges from 0.4 M to 0.6 M. For example, 0.4 M and 0.6 M is broadly used for protoplast isolation for the model plant Arabidopsis (*Arabidopsis thaliana*) and rice (*Oryza sativa*), respectively [14, 15, 19]. After testing a series of mannitol concentrations, we demonstrated that the mannitol concentration of 0.7 M is essential to achieve a high yield of areca protoplast. The released protoplasts maintain intact in the appropriate osmotic environment where mannitol concentration ranges from 0.6 M to 0.8 M. Thus, areca palm favors a relatively higher osmotic pressure of enzyme solution on protoplast yield. This is also different from the case for oil palm protoplast where they used mannitol concentration of 0.2 M in the enzyme solution [11]. The difference may result from different species, and the source material used for obtaining protoplasts.

Our results revealed that protoplast density is a major factor affecting PEG-mediated transformation efficiency for areca protoplast. This is consistent with the knowledge that protoplast density plays an important role in the highly-efficient protoplast transformation [20]. In the current study, we were able to achieve the areca protoplast transformation efficiency of nearly 50% at the protoplast density of 150 × 10^4^ cells/ml. This has been hugely improved compared to the relatively low transformation efficiency in oil palm protoplast (< 5% and 20%, respectively in two studies) [10, 11]. Nevertheless, more efforts should be devoted to the improvement of transfection efficiency for areca protoplasts. This could be possibly achieved by testing factors that often affect transformation efficiency, such as PEG concentration, amount and concentration of plasmid, and transfection time [8, 14]. In the classic system of Arabidopsis protoplast transformation, the protoplast density is adjusted to 2–5 × 10^5^ cells/ml [14, 15]. In contrast, in our platform, a higher protoplast density of 1.0–1.5 × 10^6^ cells/ml results in higher transformation efficiency compared to that of 2.5–5 × 10^5^ cells/ml. This difference could be explained in a species-dependent manner, for example the differences between sizes and permeabilities of cell membrane. However, our results are consistent with maize protoplast transformation reported by Gentzel et al. where 1.0–1.5 × 10^6^ cells/ml was used for plasmid transformation [21].

It has been reported that plant regeneration of areca palm can be successfully established through somatic embryogenesis, despite its weakness of time consuming and laborious [22, 23]. Unfortunately, there is no report on the regeneration of areca palm plants from protoplasts. Nevertheless, regeneration of viable oil palm from protoplasts have been documented [18, 24]. Future work should focus on the regeneration of areca palm plants from protoplasts and applying protoplasts for gene editing, such as CRISPR/Cas9-based genome editing.

## Conclusion

In summary, our work demonstrated that the fresh light green leaflet tissue serves as an excellent source for areca palm protoplast isolation by the direct leaf-peeling method and the mannitol concentration plays a critical role in protoplast yield. It is of potential interest to apply this so-called ‘Direct Leaf-Peeling’ method for protoplast isolation in other species in the palm family, such as oil palm and coconut. The transformation efficiency is nearly 50%, which is significantly higher than that of oil palm protoplast. To our best knowledge, this is the first efficient protocol for the high-yield isolation and efficient transformation of areca palm protoplasts. Our protoplast system offers areca palm researchers great promise for various studies in areca palm, including gene function analysis and genome editing.

## Materials and methods

### Plant material

Areca palm (*Areca catechu*) plants were regularly maintained in the outdoor experimental base of Hainan University, Hainan, China. The fresh light green leaflets from the newly-emerged leaf from the 12–30-month-old areca palm plant are selected for protoplast isolation.

### Plasmid for protoplast transformation

The green fluorescent protein (GFP)-expressing plasmid pGreen0029-GFP was a gift from Dr. Ji Li of Nanjing Agriculture University, China. The GFP expression of pGreen0029-GFP is governed by the CaMV35S promoter and nos terminator [25]. Plasmids were extracted using the Maxi Plasmid Kit Endotoxin Free (Geneaid) according to the manufacturer’s instructions. Plasmid concentration was adjusted to about 1000 ng/μl for the protoplast transformation.

### Areca palm protoplast isolation

Fresh leaflet tissue (the light green leaflet from the newly-emerged leaf) was segmented into strips of 2–4 cm in length, peeled away the lower surface using a sharp pointed tweezer and immersed in the enzyme solution [2% (w/v) cellulase R10 (Yakult Pharmaceutical Ind. Co., Ltd., Japan), 0.5% (w/v) macerozyme R10 (Yakult), 0.7 M mannitol, 10 mM CaCl_2_, 20 mM KCl, 20 mM MES and 0.1% (w/v) BSA, pH 5.7] in a Petri dish. The peeled leaves were allowed to gently shake (40 rpm on a platform shaker) in the dark overnight (12-h) at room temperature (25 ℃) for releasing protoplasts, followed by raising using the iced W5 (154 mM NaCl, 125 mM CaCl_2_, 5 mM KCl, 5 mM Glucose, 2 mM MES, pH 5.7). Areca protoplasts were then transferred to round-bottom 2 ml centrifuge tubes by passing through a 100 μm nylon mesh filter and pelleted for 3 min at 100 g. The cells were washed (100 g, 3 min) with ice-cold W5 and re-suspended in 300 μl W5. Finally, rest the protoplast solution twice on ice (one for 20 min, the other for 30 min) and resuspend the protoplasts in MMg solution (0.4 M mannitol, 15 mM MgCl_2_, 4 mM MES, pH 5.7) at a density of 1.0–1.5 × 10^6^/ml at room temperature.

### Areca palm protoplast transformation

PEG-mediated areca protoplast transformation for pGreen0029-GFP plasmid was adapted from the classic method for Arabidopsis/Tobacco protoplast transformation according to [14, 15]. More specifically, 30 μl areca protoplasts were added to 3 μl plasmid at a concentration of about 1000 ng/μl in a round-bottom 2 ml centrifuge tube and mixed gently. Finally, 33 μl freshly made PEG-calcium transfection solution (40% (w/v) PEG 4000, 0.1 M CaCl_2_, 0.2 M mannitol) were added. Samples were mixed well by gentle swirling. After 10 min incubation at room temperature, W5 was added to stop the transformation and subjected to centrifugation for 3 min at 100 g. Protoplasts were washed twice using W5 and finally stored in 50 μl of W5 (containing Ampicillin with a final concentration of 50 mg/ml).

### Protoplast yield calculation and viability assessment

Protoplasts were counted using a hemocytometer under a microscope (OLYMPUS DP80, Japan). Intact protoplasts were counted in one field, and the means were from four fields. Each experiment was performed at least in triplicate. Protoplast yield was calculated as follows:

$$\mathrm{Protoplast}\;\mathrm{yield}\lbrack\mathrm{protoplast}/\mathrm g\rbrack=\mathrm{number}\;\mathrm{of}\;\mathrm{protoplasts}\;\mathrm{yielded}\;\mathrm{in}\;\mathrm{enzyme}\;\mathrm{solution}/\mathrm{FW}\;\mathrm{of}\;\mathrm{the}\;\mathrm{leaflet}\;\mathrm{samples}\;\mathrm{used}.\mathrm{Where}\;\mathrm{FW}\;\mathrm{is}\;\mathrm{the}\;\mathrm{fresh}\;\mathrm{weight}.$$

Fluorescein diacetate (FDA) was added to 10 μl purified areca protoplasts to a final concentration of 0.05%. After incubation in the dark at room temperature for 5 min, the viability of areca protoplasts was evaluated by fluorescent microscopy. The number of protoplasts exhibiting green fluorescence (at least 80 protoplasts) under a fluorescent microscope was counted in one scope, and the means were from four scopes. Each experiment was performed at least in triplicate. Protoplast viability was calculated as follows:

$$\mathrm{Protoplast}\;\mathrm{viability}(\%)=(\mathrm{number}\;\mathrm{of}\;\mathrm{protoplasts}\;\mathrm{exhibiting}\;\mathrm{green}\;\mathrm{fluorescence}\;\mathrm{in}\;\mathrm{view}/\mathrm{number}\;\mathrm{of}\;\mathrm{total}\;\mathrm{protoplasts}\;\mathrm{in}\;\mathrm{view})\times100\%.$$

### Microscopy

The protoplasts were observed under a fluorescent microscopy (OLYMPUS DP80, Japan). For detailed visualization of GFP and chloroplast auto-fluorescence, the transformed protoplasts were observed under a confocal microscopy (ZEISS LSM 900, Germany).

### Statistical analysis

Statistical analyses were conducted using IBM SPSS Statistics. To protect against false positive (Type I) error, the Bonferroni correction at the *P* < 0.05 significance level was applied for the multiple comparisons. Data are presented as means ± SD of the mean from at least three independent experiments.

## Data Availability

All data generated in this study are included in this article.

## Abbreviations

GFP: Green fluorescent protein
CaMV35S: Cauliflower mosaic virus 35S promoter
FDA: Fluorescein diacetate
PEG: Polyethylene glycol
hpt: Hours-post transfection
BSA: Bovine serum albumin
